# Arthroscopic decompression and notchplasty for long-standing anterior cruciate ligament impingement in a patient with multiple epiphyseal dysplasia: a case report

**DOI:** 10.1186/1752-1947-2-172

**Published:** 2008-05-22

**Authors:** RK Trehan, N Dabbas, D Allwood, M Agarwal, C Kinmont

**Affiliations:** 1 Trauma and Orthopaedics, Kingston Hospital, Galsworthy Road, Kingston upon Thames, Surrey KT2 7QB, UK; 2 General Surgery, Southampton General Hospital, Tremona Road, Southampton SO16 6YD, UK; 3 Trauma and Orthopaedics, Mayday University Hospital, Croydon CR7 7YE, UK; 4 Wrightington Hospital, Hall Lane, Apley Bridge, Wigan WN6 9EP, UK

## Abstract

**Introduction:**

Multiple epiphyseal dysplasia is a genetically and clinically heterogeneous osteochondroplasia with symmetrical involvement. It is characterized by joint pain in childhood and early adulthood with early onset of osteoarthritis, mainly affecting the hips.

**Case presentation:**

We report the case of a 20-year-old man of Asian origin with multiple epiphyseal dysplasia presenting with bilateral knee pain, stiffness and instability found to be caused by bilateral anterior cruciate ligament impingement on abnormal medial femoral condyles. Bilateral staged arthroscopic notchplasty was performed successfully, resulting in subjective relief of pain, and improved range of movement and stability.

**Conclusion:**

Care should be taken not to exclude a diagnosis of multiple epiphyseal dysplasia when few of the characteristic radiographic features are evident but clinical suspicion is high. This case highlights the scope for subjective symptomatic improvement following a minimum of surgical intervention. We recommend limiting early intervention to managing symptomatic features rather than radiographic abnormalities alone.

## Introduction

Multiple epiphyseal dysplasia (MED) is a genetically and clinically heterogeneous osteochondroplasia with both autosomal dominant and recessive types, which ultimately affects the structure and integrity of collagen [1]. Altered enchondral ossification occurs in epiphyseal ossification centres of weight-bearing joints, characterised by joint pain in childhood and early adulthood with early onset osteoarthritis, mainly affecting the hips and knees [2], and usually displaying symmetrical involvement.

MED affects both sexes, and onset of symptoms usually begins early in or before the third decade of life [3], although in some forms patients experience joint pain, stiffness, gait abnormalities and retarded growth in early childhood [4]. Severity ranges from mild to severe forms, although radiographic criteria to differentiate between clinical and genetic types have been largely unsuccessful [5].

The diagnosis is based on history, physical examination and radiographic survey, although genetic linkage studies are becoming more common [4]. In early childhood, radiographic diagnosis may be difficult, but after adolescence, abnormalities become clearer [6]. The extent of knee involvement in MED has been investigated, showing radiographic abnormality in over 90% of cases [3]. The knee is the second most common joint affected after the hip [2]. In these patients, the main radiographic features before epiphyseal closure include joint space widening, genu valgum and segmentation of the epiphysis, and after epiphyseal closure characteristically include a shallow femoral trochlear groove, early osteoarthritic change, lateral tibial plateau depression and multiple free bodies [2,3]. Radiographs of the knee have also been shown to display a high positive predictive value in detecting whether a child with genetic predisposition is affected [4].

## Case presentation

A young man of Asian origin first presented at the age of 8 years with left knee pain. Initial treatment with simple analgesics failed and he presented again with ongoing knee pain that also involved the right knee. On investigation, no evidence of inflammatory arthropathy was found. A skeletal survey and bone scan were reviewed by a paediatric radiologist with expertise in children's skeletal dysplasias, who diagnosed an unusual variant of multiple epiphyseal dysplasia with relative sparing of the hips and severe changes in the knees, hands and feet. Radiographic changes in the knee joint were subtle and included genu valgum, osteoarthritic change and tibial plateau depression. There were no known affected family members and genetic testing was not carried out.

**Figure 1:**
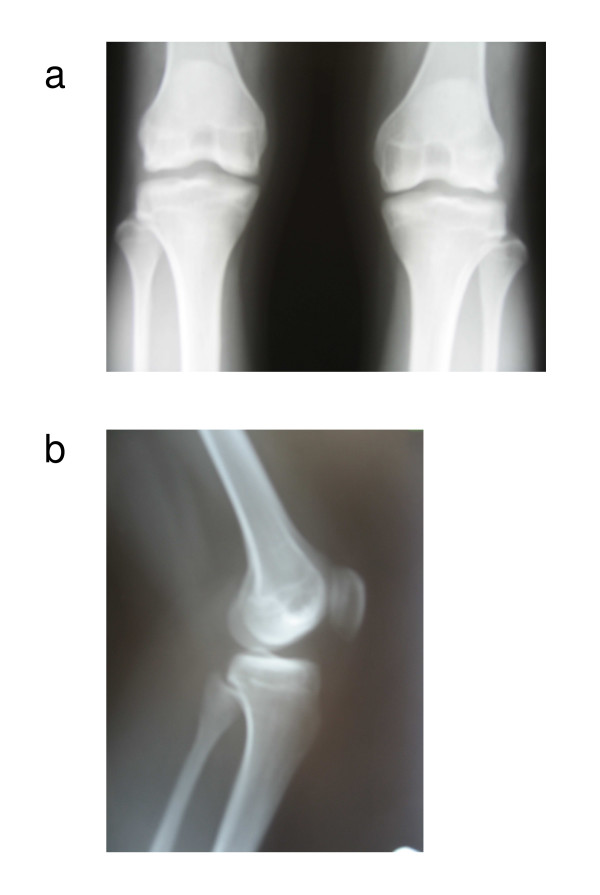
**Radiograph of the knee**. (a) Anterior-posterior view; (b) lateral view.

**Figure 2:**
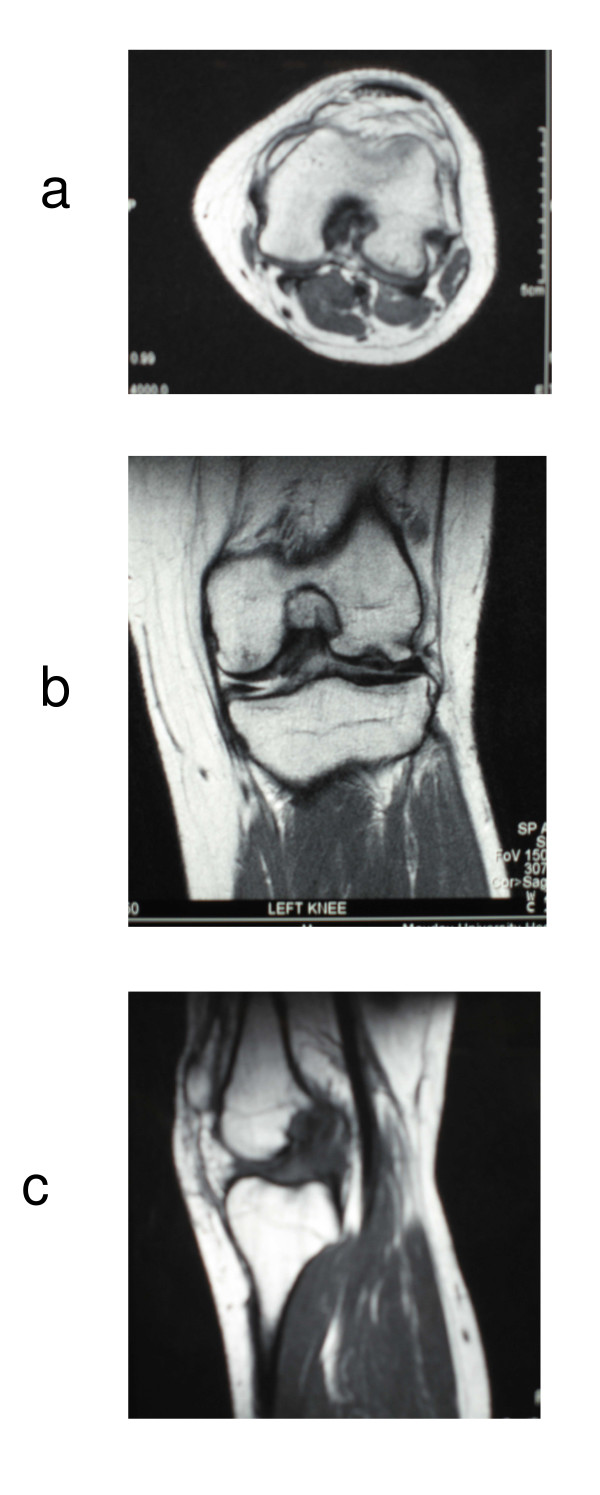
Magnetic resonance imaging of the right knee.

By the age of 16 years, the symptoms had worsened and included pain, stiffness, fixed flexion deformity of 10°, reduced range of movement with flexion restricted to 90° and subjective instability in both knees but mainly the left, as well as intermittent pain in both hips. At 18 years of age, plain imaging found no abnormality in either hip joint with both capital femoral epiphyses fused. Radiographs of the left knee at this time showed a slight depression on the joint surface of the lateral distal femoral condyle with some surrounding sclerosis, but no evidence of loose bodies (Figure 1). Magnetic resonance imaging (MRI) showed features consistent with MED (Figure 2) with an interesting finding of a dysplastic distal femur with an overgrown medial femoral condyle impinging into the notch over which the anterior cruciate ligament (ACL) was being abraded. There was also an abnormal interposition between the lateral aspect of the medial femoral condyle and the ACL.

**Figure 3:**
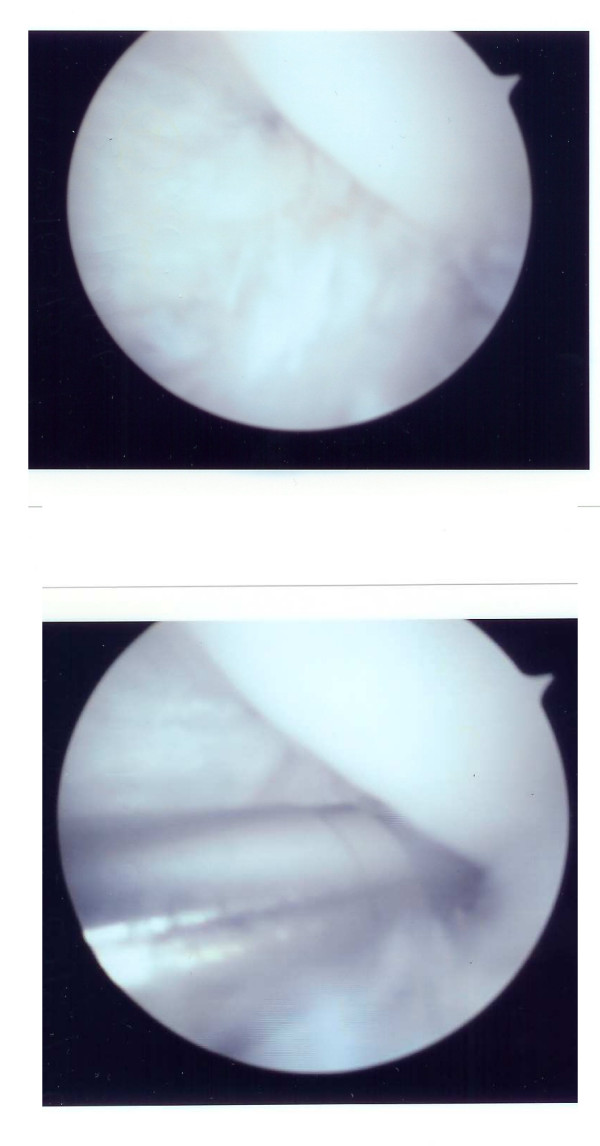
Arthroscopic images of the left knee showing an abnormal notch at the lateral aspect of the medial femoral condyle causing anterior cruciate ligament impingement.

Examination under anaesthesia confirmed restriction of motion as detailed; however, it revealed no objective abnormal anterior movement of the tibia with respect to the femur in either the left or right knee and was unable to explain the reported symptoms of instability. Arthroscopic examination of the left knee was consistent with the MRI findings, revealing an abnormal prominence of the lateral aspect of the medial femoral condyle, as well as narrowing of the notch due to an abnormal lateral femoral condyle (Figure 3). The ACL was clearly impinging within the notch and appeared to restrict the range of motion. A notchplasty was performed. The patient made an uneventful recovery, with reported reduction in pain and subjective instability. The range of joint motion had improved at 6 months to allow full extension and flexion to 110°. The Oxford knee score [7] improved from 45 pre-operation to 21 post-operation, while the SF36 [8] physical component scale improved significantly from 24.1 pre-operation to 43.8 post-operation. This improvement was maintained for 2 years; however, the patient developed symptoms in the contralateral knee. A further MRI revealed a similar appearance and a subsequent arthroscopic notchplasty of the right knee provided a comparable level of improvement with reduced pain and increase in range of movement. This improvement was maintained at the most recent review at 6 months.

## Discussion

The knee is a commonly involved site in MED, most often displaying genu valgum, shallow femoral trochlear groove, loose bodies and osteochondral defects. This case displays some of these characteristic features, but also includes bilateral medial femoral condylar abnormalities resulting in bilateral symptomatic ACL impingement.

Previous studies of MED have shown that surgical intervention is most often required in the lower limbs [9], with the knee requiring supracondylar shortening, femoral extension osteotomy, high tibial extension osteotomy, knee debridement and removal of osteophytes and posterior capsulotomy [2,9]. Disability due to MED occurs most commonly during or after the third decade, and osteoarthritis may later necessitate early total joint replacement of the hip or knee [2].

Abnormalities of the femoral condyle and intercondylar notch have been shown to be causes of ACL damage in patients with degenerative knee arthritis, and in these patients arthroscopic notchplasty was successful in improving flexion contracture, pain and instability [10]. However, no cases have been reported of this phenomenon or its surgical correction in patients with MED.

Arthroscopy has been used to successfully resect a symptomatic peri-articular osteochondroma of the distal femur [11] and is used commonly in patients suffering from MED to clear loose bodies or repair meniscal damage. In this case, we report the success of arthroscopy in treating structural ACL impingement alone resulting in marked symptomatic improvement. We speculate that the reported symptoms of instability were due, at least in part, to pseudo-instability secondary to early osteoarthritic change.

## Conclusion

MED is well known but ACL impingement leading to fixed flexion deformity and reduced range of motion have not been described in the literature to the best of our knowledge. This case highlights the scope for subjective symptomatic improvement following a minimum of surgical intervention in the form of arthroscopic decompression and/or notchplasty to resolve symptoms and to improve quality of life.

## Abbreviations

ACL: anterior cruciate ligament; MED: multiple epiphyseal dysplasia; MRI: magnetic resonance imaging.

## Competing interests

The authors declare that they have no competing interests.

## Consent

Written informed consent was obtained from the patient for publication of this case report and all accompanying images. A copy of the written consent is available for review by the Editor-in-chief of this journal.

## Authors' contributions

RKT followed-up the case and wrote the initial manuscript. ND carried out the literature search, the review of the case notes and the main drafting of the manuscript. DA helped in the review of the case notes and drafting of the manuscript. MA carried out a review of the manuscript and made the final changes before submission. CK was the senior author responsible for the case.
